# Development of the CAMUS Intra- and Postoperative Risk and Difficulty Estimation Indices Risk Prediction Tool for Estimating Peri- and Postoperative Outcomes, Including Surgical Difficulty, in Major Urological Surgery—A Protocol for a Delphi Study

**DOI:** 10.1016/j.euros.2025.04.002

**Published:** 2025-04-28

**Authors:** Christopher Soliman, Patrick Y. Wuethrich, Jochen Walz, Niall M. Corcoran, Nathan Lawrentschuk, Anna Walde, Marc A. Furrer

**Affiliations:** a Department of Urology, The University of Melbourne, Royal Melbourne Hospital, Parkville, Victoria, Australia; b Department of Anaesthesiology and Pain Medicine, Inselspital, Bern University Hospital, University of Bern, Bern, Switzerland; c Department of Urology, Institut Paoli-Calmettes Cancer Centre, Marseille, France; d Department of Urology, Western Health, Victoria, Australia; e Center for Space and Habitability, University of Bern, Bern, Switzerland; f Department of Urology, Guy’s and St. Thomas’ NHS Foundation Trust, London, UK; g Department of Urology, Solothurner Spitäler AG, Kantonsspital Olten, and Bürgerspital Solothurn, University of Bern, Switzerland

**Keywords:** Risk stratification, Surgical difficulty, Urological surgeries, Delphi method, Consensus paper

## Abstract

This protocol will outline the development of the CAMUS Intra- and Postoperative Risk and Difficulty Estimation Indices (IPRADES), a tool designed to predict perioperative outcomes and surgical complexity in major urological procedures. Its objective will be to enhance preoperative planning and risk stratification by systematically evaluating a range of patient-, organ-, and surgery-specific factors. The study will be conducted in three phases. Firstly, expert consensus will be established through a Delphi survey involving experienced high-volume surgeons to identify key parameters influencing surgical difficulty and complications. Secondly, data will be collected from the CAMUS Collaboration database and augmented with additional parameters from a prospective open cystectomy database. Thirdly, using this extensive dataset, a nomogram will be developed to evaluate the association between the identified parameters and postoperative complications, as well as to quantify their predictive values. Each parameter will be assigned a weighted significance, which will be integrated into the final risk prediction model. Statistical analyses will incorporate multivariable regression models to identify predictors of complications and mortality. The model’s performance will be evaluated through c-statistics, Hosmer-Lemeshow tests, and Brier scores, with internal validation performed via dataset splitting and bootstrap resampling. Additionally, the study will compare the predictive accuracy of universal versus procedure-specific models. The tool will not only account for patient and surgical factors, but also incorporate the influence of surgeon experience and learning curves on surgical outcomes. A web-based calculator will be developed to facilitate seamless integration into daily clinical practice, providing real-time risk assessments. Following validation, the CAMUS IPRADES tool will refine surgical planning, optimise resource allocation, and enhance patient counselling. By delivering data-driven risk assessments, it will further enable surgeon and institutional benchmarking, thereby contributing to education, training, and clinical research. Ultimately, this tool will play a pivotal role in advancing the quality and safety of urological surgeries.

## Take Home Message

Risk assessment plays a critical role in surgical decision-making, influencing patient care, resource allocation, and surgical planning. We aim to improve risk and surgical difficulty prediction to increase surgeon awareness of challenging perioperative conditions and improving patient outcomes. It also enables patients and families to better understand surgical risks.

## 1 Introduction and hypotheses

Risk assessment is a cornerstone of surgical decision-making, influencing overall patient care profoundly. Accurate risk stratification not only determines patient suitability for surgical intervention, but also guides the selection of the most appropriate treatment modality. Furthermore, it plays a pivotal role in resource allocation, surgical planning, patient counselling, postoperative follow-up, and academic reporting.

Despite the availability of numerous risk indices, each existing system has notable limitations. Most contemporary models primarily emphasise patient characteristics and comorbidities to predict morbidity and mortality. For instance, the Surgical Apgar Score (SAS) serves as a predictor of major complications or mortality within 30 d after surgery. This score has been validated across various patient populations and is a recommended tool for objectively guiding postoperative care [1]. Other risk stratification tools widely adopted in general surgery include the Acute Physiology and Chronic Health Evaluation II [2], the American College of Surgeons National Surgical Quality Improvement Program [3], the Preoperative Mortality Predictor score [4], the Surgical Risk Preoperative Assessment System [5], the Surgical Outcome Risk Tool [6], and the Physiological and Operative Severity Score for the enumeration of Mortality and morbidity (POSSUM) [7]. Table 1 provides an overview of these tools. Among these, the POSSUM score is particularly comprehensive, integrating 18 parameters encompassing patient history, biochemical data, and radiological parameters. Notably, it distinguishes itself by incorporating operative findings into its predictions. While effective for benchmarking surgical quality, POSSUM was not originally designed to guide decisions regarding surgical candidacy.

**Table 1:** Current risk estimation tools in general surgery

System	Overview	Assessed variables
Physiological and Operative Severity Score for the enUmeration of Mortality and morbidity (POSSUM)	Estimates risk for operative morbidity and mortality. Has been designed specifically for audit purposes in the general surgical patient POSSUM contains 18 variables (12 variables concern the physiological score; six variables concern the operative score). The POSSUM uses values at the time of surgery, not admission, and should not be used for trauma patients.	*Physiological score*: Age, cardiac (eg medication, peripheral oedema), respiratory (eg, dyspnoea), blood pressure, heart rate, GCS, haemoglobin, white blood count, blood urea nitrogen, sodium, potassium, ECG *Operative score*: Operative severity (eg, minor, moderate, major, or major+), number of procedures, estimated blood loss, peritoneal soiling, presence of malignancy, mode of surgery (eg, elective, emergency)
Surgical Apgar Score (SAS)	SAS can predict 30-d major complications or death after surgery. It is a 10-point score based on three intraoperative parameters.	Variables: estimated amount of blood loss, lowest heart rate, and lowest mean arterial pressure
Acute Physiology and Chronic Health Evaluation (APACHE II) score	Estimates hospital mortality. APACHE II contains 13 variables (four vitals, three oxygenation, four chemistry, three haematology, age, and GCS), using the worst value for each physiological variable within the past 24 h. A calculator will convert an APACHE II score into percentage risk of mortality	Vitals: Temp, MAP, heart rate, and resp rate Oxygenation: FiO2, PaO2, arterial pH Chemistry: sodium, potassium, creatinine, acute renal failure Haematology: haematocrit, white blood count, severe organ system insufficiency or immunocompromised patient Age GCS
American College of Surgeons National Surgical Quality Improvement Program (ACS NSQIP)	ACS NSQIP is a nationally validated, risk-adjusted, outcome-based programme to measure and improve the quality of surgical care. It estimates the chance of an unfavourable outcome (such as a complication or death) after surgery.	Age group, gender, functional status (eg, partially dependent), emergency case, ASA class, steroid use for chronic condition, ascites or congestive heart failure within 30 d prior to surgery, systemic sepsis within 48 h prior to surgery, ventilator dependence, disseminated cancer diabetes, hypertension requiring medication, dyspnoea, current smoker within 1 yr, history of severe COPD, dialysis, acute renal failure, BMI
Preoperative Mortality Predictor score	Predicts the risk of perioperative mortality in general surgery patients, is a 30-point bedside score, and contains 14 variables.	Variables: Inpatient, sepsis, poor functional status (total assistance required for all daily activities), disseminated cancer, age, comorbidities (cardiac, pulmonary, renal, and liver), steroids for chronic condition, weight loss, bleeding disorder, obesity, and “do not resuscitate status”
Surgical Risk Preoperative Assessment System	Preoperative risk assessment of postoperative mortality, overall morbidity, and six complication clusters, achieved with as many as eight (out of 28) preoperative predictor variables.	Complication clusters: infection, cardiac/transfusion, pulmonary, venous thromboembolism, renal, and neurological Top eight preoperative predictor variables: ASA class, work relative value unit, systemic sepsis (within 48 h), inpatient/outpatient, primary surgeon speciality, age, functional health status before surgery, emergency operation Remaining 20 preoperative predictor variables: transfer status, body mass index category, ventilator dependency (within 48 h), open wound with or without infection, diabetes mellitus, cigarette smoker (within 1 yr), bleeding disorder requiring hospitalisation, disseminated cancer, transfusion of more than four units PRBCs (within 72 h), >10% loss of body weight (within 6 mo), steroid use for chronic condition, ascites (within 30 d), gender, race/ethnicity, acute renal failure (rising creatinine to >3 mg/dl within 24 h), blood pressure >140/90 mmHg or taking antihypertensive medicine, severe chronic obstructive pulmonary disease, dialysis or haemofiltration (within 2 wk), dyspnoea (within 30 d), and congestive heart failure (within 30 d)
Surgical Outcome Risk Tool	Estimates the risk of death within 30 d for specific operations. Nine variables are assessed.	Variables: main group (eg urinary system and male reproductive organs), subgroup (eg, ureter), procedure description (eg, unilateral replantation of ureter into bladder), severity (eg, major), ASA class, urgency (eg, elective), cancer, age, and “thoracic, gastrointestinal or vascular surgery”

ASA = American Society of Anesthesiologists; BMI = body mass index; COPD = chronic obstructive pulmonary disease; ECG = electrocardiogram; FiO2 = fraction of inspired oxygen; GCS = Glasgow Coma Scale; MAP = mean arterial pressure; PaO2 = arterial oxygen partial pressure; PRBC = packed red blood cell; resp = respiratory; Temp = temperature.

Despite the breadth of existing indices, no risk assessment tool tailored specifically for urological surgery exists. Instead, general indices such as the Charlson Comorbidity Index (CCI) and the American Society of Anesthesiologists (ASA) physical status classification system are often relied upon to assess baseline fitness for surgical management. Although these tools are associated with morbidity and mortality outcomes in urological procedures, these were not designed with urology-specific considerations in mind, limiting their clinical applicability. Recently, frailty indices such as the Comprehensive Geriatric Assessment have gained traction for preoperative assessments; however, their utility remains confined to select populations and does not comprehensively address the broader urological patient demographic.

Another critical limitation of the existing risk stratification tools is their reliance on preoperative variables while failing to integrate intraoperative findings. Intraoperative variables, which exert a significant influence on surgical complexity and postoperative outcomes, remain largely absent from most scoring systems. For example, nephrometry scores such as RENAL, PADUA, C-index, and the RPN classification [[8], [9], [10], [11]] consider preoperative imaging characteristics, but fail to incorporate intraoperative challenges that could alter surgical risk.

To address these shortcomings, there is an imperative need for a urology-specific risk assessment tool that will incorporate both preoperative parameters (eg, prostate size and tumour extent) and intraoperative variables (eg, fibrosis and extraprostatic tumour growth). Additionally, such a tool should account for surgical approach (eg, open vs minimally invasive and transperitoneal vs retroperitoneal) and patient-specific factors that may increase surgical complexity, such as prior neoadjuvant therapies, coagulopathies, or abdominal obesity (Table 2). By integrating these intraoperative considerations, risk stratification will be refined significantly, enhancing surgical planning as well as improving patient outcomes.

**Table 2:** Proposed CAMUS intra- and postoperative risk and difficulty estimation indices (IPRADES)

CAMUS IPRADES indices								
Non–organ-specific factors impacting surgical difficulty and risk for complication								
No.	Expected/known (preoperative) parameters				Unexpected/unknown (intraoperative) parameters			
	Parameter	Surgeon		Anaesthetist	Parameter	Surgeon		Anaesthetist
		Surgical difficulty (0 = no, 1 = yes)	Risk for complication (0 = no, 1 = yes)	Risk of complication (0 = no, 1 = yes)		Surgical difficulty (0 = no, 1 = yes)	Risk for complication (0 = no, 1 = yes)	Risk of complication (0 = no, 1 = yes)
1	Diabetes				Intraoperative fluids administered (ml)			
2	ASA classification				Estimated blood loss (ml)			
3	Timing of surgery (AM, PM)				Duration of operation (min)			
4	BMI (kg/m^2^)				ECG abnormalities			
5	Anticoagulant agents				Ventilation difficulties			
6	Elective vs emergency				General overall tissue quality			
7	Previous therapy: non–organ-specific (eg, radiation)				Ooziness/tissue vascularity			
8	Charlson Comorbidity Index				Scarred tissue			
9	Prior abdominal surgical history + type of surgery (eg, bowel resection vs cholecystectomy)				Adherent tissue			
10	Nutrition status (eg, albumin, weight loss, cachexia)				Identifiable planes			
11	Age				Vision			
12	Gender				Vessel injury			
13	Steroid use for chronic condition				Intraoperative blood transfusion			
14	Smoking status				–			
15	Further parameters to be proposed by Delphi experts				Further parameters to be proposed by Delphi experts			
Unexpected/unknown (immediate postoperative) parameters								

Parameter		Surgeon				Anaesthetist		
		Risk for complication (0 = no, 1 = yes)				Risk of complication (0 = no, 1 = yes)		

Acute kidney injury (ie, eGFR decrease by 10 ml/min)								
Significant demand for analgesia (ie, opioids >50 μg)								
Targeted organ-specific factors impacting surgical difficulty and risk for complication (organ-specific scoring system) shown in an exemplary way for laparoscopic/robotic radical prostatectomy								
	Expected/known (preoperative) parameters				Unexpected/unknown (intraoperative) parameters			
	Parameter	Surgeon			Parameter	Surgeon		
		Surgical difficulty (0 = no, 1 = yes)	Risk for complication 0 = no, 1 = yes)			Surgical difficulty (0 = no, 1 = yes)	Risk for complication (0 = no, 1 = yes)	
1	Prostatic size (cc) on MRI (eg, very small or very large)				Narrow bony pelvis			
2	Size of tumour on MRI (ie, maximal diameter in cm)				Extensive intrapelvic fat tissue			
3	Numbers of preoperative biopsies				Extensive prerectal fat tissue			
4	Numbers of biopsy cores taken				Fibrosed tissue at NVB (difficulty for application of Hem-o-loks/metal clips)			
5	Type of biopsy (TRUS vs TP)				Locally advanced/infiltrative tumour (pT3b/4)			
6	Gleason score (on biopsy)				Large/flaccid bladder			
7	Previous TUR-P				Scarred/stiff bladder (neck) tissue			
8	cT stage (DRE/MRI)				Accessory obturator vein			
9	Time between biopsy and surgery (eg, 1–3, 3–6, >6 wk)				Prominent periprostatic veins			
10	Previous radiation (including dosage, eg, LDR/HDR) ± ADT				Large DVC			
11	Inguinal hernia repair (laparoscopic vs open, with/without mesh)				Intra-abdominal adhesions			
12	BMI (obesity)				Osteophyte/bone spur (symphysis)			
13	–				Friable urethral tissue			
14	Further parameters to be proposed by Delphi experts				Further parameters to be proposed by Delphi experts			

ADT = androgen deprivation therapy; ASA = American Society of Anesthesiologists; BMI = body mass index; DRE = digital rectal examination; DVC = dorsal vascular complex; ECG = electrocardiogram; eGFR = estimated glomerular filtration rate; HDR = high-dose rate; IPRADES = Intra- and Postoperative Risk and Difficulty Estimation Indices; LDR = low-dose rate; MRI = magnetic resonance imaging; NVB = neurovascular bundle; TP = transperineal; TRUS = transrectal ultrasound; TUR-P = transurethral resection of the prostate.

CAMUS IPRADES indices: To create the CAMUS IPRADES indices, four different categories of proposed parameters will be incorporated in the risk and difficulty estimation tool: preoperative (known, expected) patient-specific parameters, intraoperative (unknown or unexpected) body/patient-specific parameters, preoperative targeted organ-specific parameters, and intraoperative targeted organ-specific parameters (eg, fibrosed periprostatic tissue).

## 2 Design

### 2.1 Study design

The primary aim of this study will be to formulate and achieve consensus on pertinent pre-, peri-, and postoperative risk factors and complicating conditions by incorporating expert opinions from diverse professional backgrounds. This process will ultimately lead to the creation of a risk and surgical difficulty index ranging from 0 (indicating no risk of complications and the lowest grade of difficulty relative to a standard procedure) to 100 (indicating death or procedure abandonment due to an excessively high level of difficulty). The authors acknowledge that no surgical procedure will be entirely devoid of risk; however, the adoption of a 0–100 scale will provide a pragmatic framework for risk stratification.

**Fig. 1:**
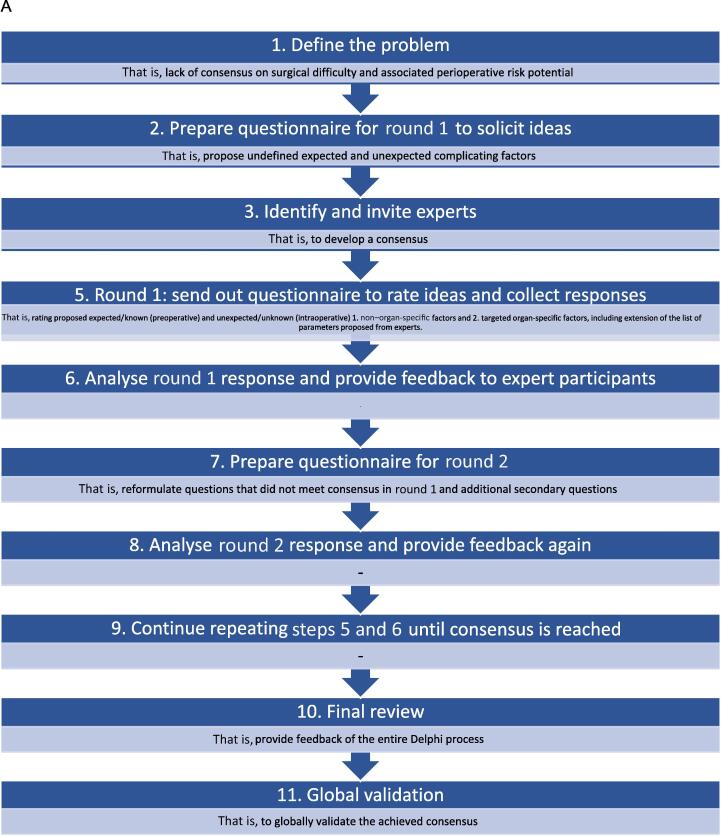

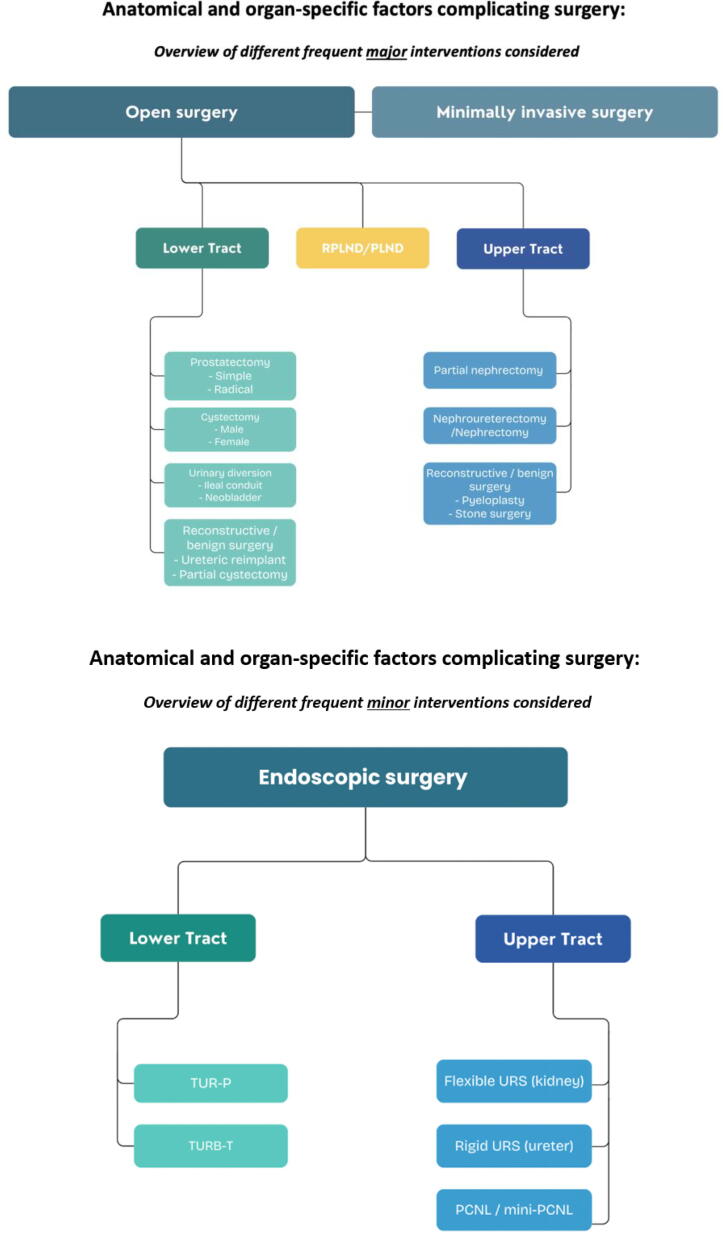
(A) The 11 steps of the Delphi method to achieve global consensus on perioperative risk and difficult estimation. (B) Overview of organ- and approach specific interventions for which targeted organ-specific factors impacting surgical difficulty and risk for complication (organ-specific scoring system) will be defined. PCNL = percutaneous nephrolithotomy; PLND = pelvic lymph node dissection; RPLND = retroperitoneal lymph node dissection; TUR-B = transurethral resection of the bladder; TUR-P = transurethral resection of the prostate; URS = ureteroscopy.

The Delphi method, a well-established technique for developing consensus-based best practice guidelines [[12], [13], [14], [15], [16], [17], [18], [19]], will serve as the foundation of this study. This scientifically validated group communication process will encompass 11 structures [13,20], which will be delineated in detail within the study methodology (see Fig. 1A).

A summary of the Delphi study approach is presented in Table 3, encompassing key aspects such as method selection, expert participant nomination, invitation and follow-up protocols, sample size considerations, response rate, consensus definition, and selection of the steering committee. Expert participants will include high-volume local and international urologists, general surgeons, and consultant anaesthetists, with a particular emphasis on those specialising in urological critical care. These participants will be identified through the CAMUS Collaboration databases and trial coordinators, and will be contacted via e-mail. All invited experts will be encouraged to complete all Delphi rounds. Round 1 will remain open for 4 wk, with weekly reminders issued to ensure engagement. The study will aim for a minimum sample size of 50 participants, comprising 20 urologists, ten general surgeons, and 20 anaesthetists. In cases of participant withdrawal, follow-up inquiries will be conducted to ascertain the reasons for discontinuation.

**Table 3:** Study approach

#	Section	Question
1	Delphi method	Eleven steps of the Delphi study (see Fig. 1).
2	Selection of nominated expert participants	Local and international high-volume urologists, general surgeons, and consultant anaesthetists are invited to complete the survey. Primarily urological experienced critical care specialists will be targeted.
3	Invitations and reminders to Delphi participants	Participant experts will be contacted via e-mail (obtained via CAMUS databases and trial coordinators). All participants will be invited to complete all Delphi rounds. Round 1 will be closed after 4 wk. Reminders will be sent every 7 d.
4	Sample size and response rate for the Delphi study	Minimum sample size of 50 participants (20 urologists, ten general surgeons, 20 anaesthetists) will be targeted. In case of withdrawal, participants will be contacted to identify the reason for withdrawal.
5	Defining and achieving final consensus	Consensus will be defined, a priori, as majority agreement (≥75%) of participant response. If consensus is not reached, then subsequent Delphi rounds (minimum of two, maximum of five) will be performed under the guidance of the steering committee.
6	Steering committee selection	Committee members will be selected primarily from our team of investigators, as well as several highly experienced specialists (urologists, general surgeons, anaesthetists, methodologists, and radiologists [for assessment/discussion of radiologically evident risk factors, eg, clinical staging]) in surgical reporting.

Consensus will be defined, a priori, as a majority agreement of ≥75% among participants [15,21,22]. If consensus is not achieved, additional Delphi rounds (ranging from a minimum of two to a maximum of five) will be conducted under the guidance of the steering committee. This committee will be composed primarily of study investigators and highly experienced specialists, including urologists, general surgeons, anaesthetists, radiologists (for the assessment of radiological risk factors such as clinical staging), and methodologists specialising in surgical reporting.

In the initial phase, the most common uro-oncological procedures will be evaluated to establish organ-specific correlations with surgical difficulty and peri- and postoperative risk profiles (see Fig. 1B). These procedures will include standard radical prostatectomy (RP), transperitoneal partial nephrectomy (PN), nephroureterectomy/nephrectomy, and radical cystectomy (RC; both male and female) with urinary diversion, encompassing ileal conduit and orthotopic neobladder formation.

Should these indices demonstrate clinical applicability through prospective validation, further evaluations of additional major and minor interventions will be undertaken in subsequent Delphi processes (Fig. 1B).

Ethics approval for this study has been granted through the Research Governance and Quality Assurance Team at Epworth HealthCare (Epworth ID: EH2021-708). Furthermore, the study has been registered with ClinicalTrials.gov PRS (NCT04976946) as of August 12, 2021.

### 2.2 Study aims

The final Delphi document will aim to synthesise expert consensus and provide guidance in areas of on-going debate within the field of urological surgery. Its findings will be of significant relevance and potential applicability to other surgical disciplines, ensuring the dissemination of optimised guidance to clinicians and patients alike. Ultimately, these position statements will be incorporated into internationally recognised guidelines issued by urological associations.

The primary objective of this study will be to develop a peri- and intraoperative risk prediction index and scoring system to estimate and quantify surgical difficulty, as well as to construct targeted, organ-specific scoring systems based on pre- and intraoperative factors. Similar to the existing renal scoring systems [8,9,11], the objective will be to establish a checklist-based framework to assist in surgical planning, case selection, and stratification of procedures based on their suitability for teaching purposes (see Fig. 1B).

The main objectives of the study will be the following (Table 4):

1. To predict intraoperative (during intervention), perioperative (in-hospital period), and postoperative (within a defined period after intervention) outcomes by considering both pre- and intraoperative factors
2. To predict intra-, peri-, and postoperative outcomes based solely on preoperative factors, thereby optimising resource allocation and enabling the identification of appropriate teaching cases prior to surgery
3. To define surgical difficulty as an independent metric for the comparative analysis of intra-, peri-, and postoperative outcomes across surgeons and procedures. This will necessitate the delineation of parameters contributing to surgical complexity from both an anaesthetic and a surgical perspective. Consideration will be given to anaesthetic and surgical case volume, frequency of procedures over a given timeframe, and individual learning curves, as these factors will influence both case complexity and the risk of complications
4. To develop multiple indices for surgical risk and difficulty estimation, which will be calculated both preoperatively and immediately postoperatively

**Table 4:** Study aims

#	Aim
1	To predict intra-, peri-, and postoperative outcomes by considering pre- and intraoperative factors
2	To predict intra-, peri-, and postoperative outcomes by considering preoperative factors only, to optimise the allocation of critical care resources or defining teaching-appropriate cases before surgery starts
3	To determine surgical difficulty as a separate matter to judge surgical performance by improving the comparison of intra- and postoperative outcomes between surgeons and between different interventions
4	To define parameters that make certain operations (and approaches) for anaesthetists and urologists more difficult
5	To create several indices (surgical risk and difficulty estimation) that are calculated before and immediately after surgery

The surgical difficulty and risk estimation tool will integrate five categories of patient-specific parameters (Table 2):

1. Preoperative (known, expected) patient-specific characteristics and conditions (eg, body mass index [BMI], ASA score, sex, prior abdominal surgery, and comorbidities)
2. Intraoperative (unknown or unexpected) body/patient-specific characteristics and conditions (eg, bleeding tendency, restricted operative space due to a narrow bony pelvis, or extensive adhesions)
3. Preoperative targeted organ-specific parameters (eg, tumour size and location, organ anatomy and morphology, or a history of prior interventions on the targeted organ such as biopsies or transurethral resection of the prostate preceding prostatectomy)
4. Intraoperative targeted organ-specific parameters (eg, fibrosed periprostatic tissue or tumour extension beyond the targeted organ).
5. Unexpected/unknown postoperative conditions prior to discharge or within 24 h (eg, acute kidney injury, quantified by a decrease in glomerular filtration rate [ml/min])

Surgical and anaesthetic parameters will be evaluated independently to account for the influence of case volume and individual learning curves on surgical complexity and risk. The key variables will include the total case volume, frequency of procedures performed within a defined period, and complexity of prior cases. Additionally, institutional case load will be considered when estimating perioperative morbidity risk (eg, in tertiary referral centres).

## 3 Protocol overview

### 3.1 Study development and structure

The Delphi survey will be developed using the browser-based REDCap software, chosen for its ease of use, efficient distribution, and ability to maintain participant anonymity. The survey will be structured into five distinct parts:

1. *Part 1*: Will collect participant demographic details (eg, age, location, and experience) to assist with quality assurance.
2. *Part 2*: Will evaluate how experts identify parameters impacting surgical difficulty and risk factors. This section will aim to gather information on participants' experience and opinions regarding factors influencing surgical difficulty and peri- and postoperative complication risks, as well as their perspectives on the strengths, weaknesses, and applicability of existing perioperative risk prediction tools.
3. *Part 3*: Will focus on expected/known (preoperative) non–organ-specific factors that impact surgical difficulty and complication risk, addressing how these factors contribute to perioperative risk estimation.
4. *Part 4*: Will examine unexpected/unknown (intraoperative) non–organ-specific factors, organ-specific parameters affecting surgical difficulty and complication risk, and immediate postoperative conditions that may influence peri- and postoperative risk estimation.
5. *Part 5*: Will collect participant identification and consent.

Each section will contribute distinct data, which will be elaborated upon further in the survey outline. For a comprehensive breakdown of the description, content, aims, and rationale behind the five parts of the Delphi survey, see Table 5. To ensure quality control, the questionnaire will be developed in collaboration with methodologists and reviewed by experienced urologists.

**Table 5:** Description, content, aims, and rationale of the five parts of the Delphi Survey

Part	Description	Content, aims, and rationale
1	Participant demographic details	Basic participant demographic information (eg, age, location, experience) to assist in quality assurance
2	How to identify parameters impacting surgical difficulty and risk factors	Information regarding gauging participant experience in opinions of potential factors impacting surgical difficulty and peri- and postoperative risk for complications; and opinion on the meaningfulness, strengths, and weaknesses of pre-existing perioperative risk prediction tools
3	Non–organ-specific factors impacting surgical difficulty and risk for complication	Achieving consensus on patient-specific characteristics and conditions that may affect the difficulty of surgery and risk stratification
3A	Expected/known (preoperative) non–organ-specific parameters	Defining preoperatively existing patient-specific parameters (eg, body mass index, ASA score, biological sex, prior abdominal surgery, diabetes)
3B	Unexpected/unknown (intraoperative) non–organ-specific parameters	Defining patient-specific parameters that occur unexpectedly or become apparent during the operation (eg, bleeding tendency or extensive adhesions)
3C	Unexpected/unknown (postoperative) conditions occurring prior to discharge or max within 24 h (eg, acute kidney injury quantified by GFR decrease [%])	Defining immediate postoperative parameters that might have an impact on morbidity and mortality (eg, acute kidney injury quantified by GFR decrease [ml/min])
4	Targeted organ-specific factors impacting surgical difficulty and risk for complication (organ-specific scoring systems)	Obtaining consensus on patient-specific characteristics and conditions that may affect the difficulty of surgery and risk stratification
4A	Expected/known (preoperative) targeted organ-specific parameters	Defining preoperatively existing targeted organ-specific parameters (eg, size and location of the tumour, anatomy and morphology of the organ)
4B	Unexpected/unknown (intraoperative) targeted organ-specific parameters	Defining patient-specific parameters that occur unexpectedly or become apparent during the operation (eg, fibrosed periprostatic tissue, cancer extension beyond the confines of the targeted organ)
5	Participant identification and consent	Requesting the full name, preferred title, and professional affiliations of each participant of the Delphi survey for quality assurance and further ensuring that participants are compensated for their time with PubMed listed collaborative authorship

ASA = American Society of Anesthesiologists; GFR = glomerular filtration rate.

### 3.2 Assessment of risk and difficulty factors

Risk factors affecting surgeon performance will be rated exclusively by surgeons, whereas those impacting the anaesthetist will be assessed solely by anaesthetists. Risk factors influencing both surgical and anaesthetic outcomes will be evaluated by both groups. Surgeons will assess parameters related to surgical difficulty and complication risk, while anaesthetists will focus on factors contributing to the risk of complications.

To enhance accessibility, the risk prediction index will primarily utilise routinely available preoperative patient characteristics and easily quantifiable intraoperative conditions. Each variable will be assigned a numerical value, with higher values indicating greater significance in determining outcomes.

As peri- and postoperative complications arise from both surgical and anaesthetic risk factors, input from both disciplines will be essential in developing the index.

To evaluate the association between proposed parameters and complications, and to estimate their likelihood, a nomogram will be constructed using data from the CAMUS Collaboration database, supplemented with parameters from a prospective open cystectomy database at the University of Bern. The likelihood of complications will be critical, as each parameter will be assigned a specific weight to be incorporated into the risk prediction score.

Institutional databases will be utilised to create a model predicting the impact of certain parameters on peri- and postoperative complications at 90 and 360 d after surgery. Additionally, organ-specific scoring systems for bladder, prostate, kidney, and testicular cancer will be developed using a nomogram and institutional databases.

### 3.3 Identification and description of factors

In the initial phase of the study, risk-predicting parameters will be defined using data from the CAMUS Collaboration cystectomy database, which will include pre- and intraoperative variables sourced from 178 centres worldwide. Additional parameters from a prospective open cystectomy database maintained by the Urology Department at the University of Bern will further enhance data collection. The difficulty estimation score will be developed and validated using these data, supplemented by prospectively collected parameters detailed in round 1 of the study.

The RC cohort will be selected as the primary focus due to its high complication rate and clinical relevance. In subsequent phases, the scoring system’s performance will be assessed in various patient cohorts who have undergone RP, PN, radical nephrectomy (RN), radical nephroureterectomy (RNU), and retroperitoneal lymph node dissection (RPLND).

The first Delphi round will involve expert consensus to identify parameters influencing peri- and postoperative morbidity, mortality, and surgical difficulty. For example, parameters relevant to RP will be outlined in Supplementary Tables 1 and 2. Experts will be guided by the results derived from nomograms. Organ-specific factors, as part of the organ-specific scoring system, will also be examined for procedures such as RC, RP, PN, RN, RNU, and RPLND, with further details provided in Supplementary Table 3.

To assess the impact of these parameters on both difficulty and risk, a comprehensive description of the associated conditions and characteristics will be required. These parameters will be categorised according to a five-level scale of difficulty: easiest, intermediate easy, standard, intermediate difficult, and most difficult. For instance, preoperative BMI ranges will be classified as follows: BMI 18–22 kg/m^2^ will correspond to the easiest grade, while BMI >40 kg/m^2^ will be categorised as most difficult. Similarly, for organ-specific factors such as prostate size in RP, a prostate volume of 20–30 cc on magnetic resonance imaging will represent the easiest level of difficulty, whereas a volume exceeding 200 cc will be classified as most difficult.

Notably, multiple conditions may be associated with the same level of difficulty. For example, multiple prior open procedures or laparoscopic surgeries involving mesh insertion may be considered of intermediate difficulty.

In the second Delphi round, the complexity range (difficulty/risk) associated with each identified factor will be refined further. Experts will evaluate the significance of each factor concerning morbidity, mortality, and surgical difficulty using a numerical scale. Separate ratings will be assigned for risk estimation and surgical difficulty, ranging from 0 to 10. A rating of 0 will indicate no impact on surgical difficulty or risk, whereas a rating of 10 will signify a strong association with significant surgical difficulty and a near-certain major complication. For example, the difficulty of tissue plane dissection might be rated as follows: completely adherent planes (9), normal planes (5), and easily identifiable and dissectable planes (0).

Through the Delphi process, consensus will be reached regarding key parameters, their specific conditions and manifestations, and their quantified impact (0–10) on risk and surgical difficulty. These consensus parameters will be integrated into the risk and difficulty indices, which will be calculated automatically.

In clinical practice, surgeons will assess these parameters both preoperatively and postoperatively. Rather than assigning numerical values, surgeons will evaluate these according to a structured risk and difficulty scale (0–5), as outlined above. This approach will streamline decision-making and facilitate practical application of the indices.

### 3.4 Clinical scenario for rating general and targeted organ-specific parameters in RP

Preoperatively known general and targeted organ-specific factors will be entered into a computer system or calculator. Their impact on risk and surgical difficulty will be assessed and recorded separately.

For instance, a prostate size of 40 cc will correlate with a surgical difficulty rating of 2 (intermediate easy), while prior laparoscopic procedures involving tissue resection (eg, bowel anastomosis) or mesh insertion will be classified as standard difficulty (grade 3).

In terms of peri- and postoperative risk estimation, well-controlled diabetes will be assigned a grade 1 condition.

As outlined previously, preoperative risk and difficulty estimation for RP will be conducted based on the general and organ-specific factors identified preoperatively. Immediately postoperatively, the surgeon will update risk and difficulty estimates based on intraoperative conditions. For example, if tissue planes are classified as partially adherent, partially identifiable, and dissectible, a standard difficulty rating (grade 3) will be recorded. If significant bleeding occurs without clotting but with intact vision, a grade 4 (intermediate difficulty) condition will be noted. For intraoperative parameters influencing peri- and postoperative risks, blood loss ranging from 100 to 500 ml will be considered a grade 2 condition.

To further refine the morbidity and mortality risk estimation following RP, postoperative conditions occurring prior to discharge or within 24 h will be entered into the system. A decrease in glomerular filtration rate by 60% will be classified as a grade 4 condition.

### 3.5 Weighing of factors

Once consensus has been reached on the parameters for developing the risk and difficulty stratification tool, both surgeons and anaesthetists will assign weights to each parameter. These weights will be represented as ws + wa = (weight of the surgeon × weight of the anaesthetist), where “ws” denotes the weight assigned by the surgeon and “wa” the weight assigned by the anaesthetist. The weighting will depend on whether the parameter is assessed by both specialists or exclusively by one discipline.

Parameters will be assigned separate weights for surgical difficulty and risk of complications. The weighting system will be structured so that parameters with a low likelihood of complications, those associated with less severe complications (eg, conditions manageable through perioperative interventions), or those resulting in minimal intraoperative difficulty are assigned lower values. Conversely, parameters with a higher likelihood of complications, greater severity, or increased intraoperative difficulty will be allocated higher values.

### 3.6 Development of the risk and difficulty stratification index

Following the consensus phase, the next step is to develop the formula for the risk prediction index. In this phase, the weights assigned by both surgeons and anaesthetists (ie, the median reference value) will be incorporated into a formula, which will then be normalised to a value ranging from 0 to 100. Similar to the Comprehensive Complication Index (CCI) formula [23,24], variables may be included to ensure that the model assigns higher values for multiple and severe complications while yielding lower values for single and minor peri- and postoperative complications. This approach will enhance the precision of differentiating between the severity and impact of pre- and postoperative risk factors. Organ-specific scoring systems will be developed following the same methodology.

Based on the outcomes of the Delphi process, we will determine whether a single formula or index is sufficient to address all three objectives outlined or whether multiple indices are required. Specifically, one index may be necessary to assess preoperative factors alone (eg, to enhance decision-making, optimise the allocation of critical care resources, or designate surgeries as teaching cases), while another index may be needed to incorporate both pre- and intraoperative factors for broader objectives. If surgical difficulty requires an independent calculation, a third distinct index may be warranted.

As highlighted previously, a surgeon’s learning curve—defined by a cumulative case volume—is a critical factor. This curve will be stratified into experience-based stages: 0–20 cases, 21–50 cases, 51–100 cases, 101–250 cases, 251–500 cases, and >500 cases. Proficiency typically plateaus after approximately 500 cases.

The assertion that surgical proficiency plateaus after around 500 cases is supported by studies analysing learning curves in complex surgical procedures. Research using a cumulative sum (CUSUM) analysis and other models has demonstrated that significant improvements in performance metrics—such as operative time, complication rates, and technical outcomes—tend to stabilise after approximately 500 cases, particularly in highly complex surgeries [25,26].

This stratification serves two purposes: (1) it allows the estimation of how increasing surgical experience reduces case difficulty and risk, and (2) it helps determine whether a procedure is suitable for a teaching setting.

It is essential to differentiate between the absolute and relative risks within this framework. Certain patient-specific factors, such as prior radiation therapy, inherently elevate the absolute risk and case difficulty. However, a highly experienced surgeon (eg, one with >500 completed cases) can mitigate these challenges in relative terms. Consequently, the calculated risk and difficulty index for a given case may be adjusted downward when performed by an experienced surgeon, incorporating their proficiency into the assessment model.

### 3.7 Data collection

The CAMUS Collaboration has established four independent outcome databases for patients undergoing open or minimally invasive RC, RP, PN, RN, RNU, and RPLND, This collaborative study has collected various pre-, intra-, and postoperative outcome variables, along with complication data (including complication type, intervention, and grading), to evaluate their potential as predictors for intra- and perioperative risk estimation. Additionally, a comprehensive single-centre cystectomy database from the University Hospital Bern (*n* = 1550) is included. In more detail, the CAMUS database includes a diverse patient population from various centres, reflecting a wide range of clinical scenarios and surgical outcomes. To minimise the potential selection bias, the exclusion criteria were restricted to patients with ≤10% missing data and by age factor, allowing the inclusion of all patients aged 18 yr and older. This approach ensures a representative cohort and enhances the generalisability of our results.

The required sample size was estimated using a power-based method for logistic regression models, considering the number of predictor variables ranging from 10 to 30 (refer to section 6.1 for more information). The calculations were performed using the pwr package [1] in the statistics software R, version 4.4.2 [2], utilising effect sizes based on assumed odds ratios of 1.2 (weak effect), 1.5 (moderate effect), and 2.0 (strong effect). The required sample size is reached when a statistical power of at least 80% is achieved at an alpha level of 0.05. To enable internal validation (see section 6.3), the final sample size was increased by 50%, ensuring sufficient data for both model training and validation. Consequently, the most extreme case—30 predictors with a weak effect size (odds ratio = 1.2)—required a sample size of 1180, which is well below the available sample size.

Patients eligible for inclusion are aged 18 yr or older.

Data collection has been conducted by trained and audited registrars, consultants, and study nurses at each participating hospital, ensuring standardised data definitions across all institutions. Missing data have been managed using imputation, specifically employing the Buck’s method. Patients with missing values for critical variables—such as the date of postoperative complication-intervention events, Clavien-Dindo complication (CDC) grade, or death within 90 d—have been excluded from the analysis.

For inclusion in the initial patient cohort (cohort 1), all patients who underwent open or minimally invasive RC and urinary diversion between 2000 and 2021 were selected. Cohort 2 includes all patients who underwent open or minimally invasive RP between 2000 and 2020. Cohort 3 comprises all patients who underwent PN, RN, RNU, and RPLND. All variables from the CAMUS database have been included in the analysis.

Certain variables were adjusted for analytical purposes, such as categorising renal function into grades 1–5 and grouping intervention events (eg, intensive care treatment) into broader categories with subgroups (eg, cardiac and pulmonary intensive care). These adjustments were made to establish preoperative comorbidity categories and transform non-normally distributed variables. The primary study outcomes are the CDC/CAMUS grade or death within 90 d and the Comprehensive Complication Index (CCI).

Preoperative (expected/known) risk and difficulty factors used to calculate patient-specific risks and surgical difficulty were selected a priori based on their predictive value, routine availability to the surgeon before the operation, and clinical face validity. Similarly, intraoperative (unexpected/unknown) risk and difficulty factors were selected based on their predictive value, the surgeon’s expertise, and clinical validity. Immediate postoperative factors that refine risk estimation follow the same methodology.

All preoperative, intraoperative, and immediate postoperative factors will be evaluated using the Delphi method, as described previously.

## 4 Statistical analysis

### 4.1 Statistical analysis and development of the score

All statistical analyses will be conducted using R packages. Initially, a univariate analysis will be performed to examine the relationship between each pre- and intraoperative variable in the database and the primary outcomes. Variables with *p* ≤ 0.20 and ≤10% missing data will be considered for inclusion in a multivariable model, to ensure sensitivity and prevent the exclusion of potentially relevant factors. Among collinear variables, selection will be based on *p* values and their potential use in constructing an outcome score.

In the multivariable analysis, intraoperative variables will be entered independently and in combination with preoperative variables into a logistic regression model. Both forward and backward stepwise selection procedures will be employed (with entry criteria *p* ≤ 0.10 and inclusion criteria *p* ≤ 0.05) for outcomes of interest. The final model will be selected based on the Akaike Information Criterion (AIC). Variables that independently predict major complications or death will be considered as candidates for the risk index score. Alternative combinations of variables for the risk index score will be compared, emphasising simplicity, clinical safety, and predictive power.

After determining the final variables for inclusion, the magnitude of the β coefficients from the regression equation will be used to assign weights to each variable. Depending on the number of variables included, the score will be represented as either a 10-point or a 20-point scale. The intervals and cut-offs for each point level will be designed to ensure that a 1-point increase in the score corresponds to a proportional increase in the odds of a complication. The values for each cut-off will then be rounded to clinically relevant figures that can be recalled easily by surgeons. For instance, operation duration will be valued as follows: 0 points for ≤120 min, 1 point for 120–180 min, etc.

Logistic regression will be used to examine the relationship between major complications/death, and the risk index score (treated as an ordered categorical variable based on predefined categories) and its components across the three patient cohorts. Model discrimination will be assessed by calculating c-statistics (area under the receiver operating characteristic curve). For model calibration, the Hosmer-Lemeshow (HL) goodness-of-fit test will be used to compare predicted versus observed outcomes between the two cystectomy cohorts. In the final cohort, differences in complication rates across individual scores will be compared using chi-square tests. Additionally, using morbidity (complicated/uncomplicated) and mortality (dead/alive) as dichotomous dependent variables, multiple logistic regression models will be derived. Overall model significance will be assessed via the model chi-square statistic, and differences between observed and expected outcomes will be evaluated using the HL test.

To develop the surgical difficulty index, pre-, intra-, and postoperative parameters and outcome measures will be determined by experienced high-volume surgeons (≥1000 major interventions and >10 yr of surgical experience) using the Delphi method. Pre- and perioperative parameters that potentially increase operative difficulty and risk will be assessed using a 5-point scale, as shown in Supplementary Tables 1–3. Primary outcome measures for surgical difficulty will include operation duration, blood loss and transfusion, use of haemostatic agents, and the surgeon’s judgement (on a scale from 0 to 100).

The performance of the universal surgical risk calculator, which includes multiple specialities and hundreds of procedures, will be compared with the performance of our risk calculators, specifically programmed for this score development. This comparison will be made for individual procedures (eg, RC and RP).

Alternatively, to create risk prediction models, random-intercept, fixed-slope hierarchical models (using SAS GLIMMIX) will be employed. These models account for case clustering within hospitals and impose an empirical Bayes-type shrinkage adjustment. As a result, both universal and procedure-specific surgical risk calculators will be developed. The performance of the universal versus procedure-specific models will be evaluated using three metrics: the c-statistic, the HL statistic (in a revised graphical form), and the Brier score.

Prediction models for mortality, CCI, blood loss, operating time, and other outcomes will be developed using logistic regression with stepwise selection based on the AIC. Cumulative C-indices will be recorded at each step of model development.

Given the HL test’s sensitivity to sample size, graphs will be constructed based on sequential groups with equal observed events rather than equal patient numbers (thus, low-risk groups will have more patients than high-risk groups).

For evaluating risk prediction accuracy and comparing the universal and procedure-specific surgical risk calculators, the Brier score will be considered the most appropriate measure of model performance. Results from 2 yr of data used for model development will be compared with those from the 3rd year of data used for validation.

### 4.2 Surgeon risk adjustment

Given that the surgical difficulty and peri- and postoperative complication risks estimated by the calculator may not encompass every potential comorbidity, an ad hoc provision will be introduced, allowing surgeons to adjust the estimated risks reasonably. Recognising the inherent uncertainty (confidence interval) around the calculated risks, clinicians will be permitted to modify the risk and difficulty estimates within the confidence interval for each outcome measure. Surgeons will be allowed to increase the estimated risks by up to 30 points (on a scale of 0–100) if deemed necessary.

To evaluate the relative value of generic versus surgery-specific risk models, surgery-specific models will be developed for the three most common surgical specialities in the database (RC, RP, and PN). The C-indices, HL analyses, and Brier scores for the generic model will then be compared against those of the surgery-specific models applied to the same patient subgroups defined by subspeciality.

By creating a single model applicable across a wide range of surgical outcomes and difficulties, the aim is to streamline the preoperative risk assessment process. However, it is acknowledged that, in certain circumstances, surgery-specific models may yield more precise risk estimates. This suggests that the optimal strategy may involve a balanced approach, incorporating the simplicity of a generic model while maintaining the accuracy of speciality-specific models where appropriate.

### 4.3 Internal validation

Internal validation of the prediction models will be conducted by randomly splitting the analytic dataset into two subsets: a developmental dataset (two-thirds of the total data) and a validation dataset (one-third of the total data). The C-indices, HL analyses, and Brier scores from the logistic regression analyses will be compared between the developmental model applied to the developmental dataset and the same model applied to the validation dataset.

The use of stepwise, forward-selection logistic regression has faced criticism, particularly in small samples, regarding variable selection and the accuracy of the coefficients and standard errors. However, such criticisms are less relevant when applied to the large sample sizes present in this study. To further test the robustness of the model, bootstrap resampling will be employed for the postoperative mortality model. Full-variable logistic regression models will be performed on each bootstrap sample. The variables will then be ranked by the magnitude of the Wald χ^2^ statistic, and the average rankings from the bootstrap samples will be compared with the order of entry of variables during the forward selection stepwise regression analysis. As such, all steps described above aim to minimise overfitting and improve predictive accuracy.

### 4.4 External validation

Once the indices have been developed and following internal validation, external validation in a prospective multicentric validation cohort is crucial for ensuring the generalisability, applicability, and robustness of the CAMUS IPRADES model. In particular, the investigators of this multilevel study aim to validate the model with a multicentric dataset from high-volume urological centres and are actively exploring collaborations within the CAMUS network and beyond to support this next phase.

### 4.5 Development of a calculator

A calculator is being developed and integrated into an electronic database to simultaneously compute all the indices mentioned above using the same input data. Consequently, even if multiple indices are required, the parameters will need to be entered into the system only once. As a result, the time required to calculate a single score will be the same as that needed to calculate all the three scores.

## 5 Summary

### 5.1 Discussion

The ultimate goal of risk prediction is to raise awareness among surgeons regarding challenging perioperative conditions, thereby preventing negligence and reducing the risk of complications. Additionally, it helps patients and their families better understand the implications of complex surgical procedures. By estimating individual risks and difficulties for each patient, this approach facilitates stratification of surgeries, helping in determining which procedures may be suitable for training or teaching purposes.

Although clinical judgement based on pre- and intraoperative factors is crucial, it alone is insufficient for predicting adverse outcomes following surgery [27]. Consequently, various perioperative risk prediction tools, such as risk stratification calculators, have been developed and validated to identify high-risk patients [28,29]. Emerging evidence continues to demonstrate that proper risk assessment improves patient outcomes significantly. These tools rely on multiple patient-specific variables and mathematical models calibrated using large datasets, providing a quantitative assessment of risk.

These variables can also be used to compare expected and observed outcomes across different surgeons, allowing for individual performance tracking as well as comparisons between various centres and regions. However, despite their potential, most of these tools are not yet implemented routinely, as none meet all the criteria for an ideal scoring system.

All contributing factors should ultimately be synthesised into a comprehensive scoring system comprising both a “targeted organ-specific subscore”, which reflects preoperative parameters, and an integrated score that combines pre- and intraoperative considerations. This consolidated scoring system would serve as a vital component of an overarching risk assessment tool.

The surgical difficulty and risk estimation framework should encompass five distinct categories of patient-specific parameters: (1) preoperative patient- or body-specific characteristics and conditions (known and expected), (2) intraoperative patient- or body-specific factors (unknown or unexpected), (3) preoperative organ-specific parameters (known and expected), (4) intraoperative organ-specific parameters (unknown or unexpected), and (5) postoperative conditions (unknown or unexpected) observed before discharge or within the first 24 h.

Furthermore, factors extending beyond patient-specific considerations—such as anaesthetic and surgical case volumes, as well as the progression of individual learning curves—must be incorporated into tools designed to estimate surgical difficulty and associated risks. These components play a pivotal role in mitigating procedural complexity and minimising the likelihood of complications.

Learning curves, which delineate the correlation between cumulative experience and performance outcomes, offer valuable insights for evaluating surgeon competency, optimising surgical performance, and developing health care education and training strategies. Importantly, these can also predict perioperative morbidity risks and the overall complexity of surgical procedures [26,30].

Individual variability in surgeons’ prior experience and expertise introduces heterogeneity in baseline performance levels. The predominant mode of skill acquisition—active learning supplemented with feedback—typically results in incremental improvements. However, these gains may be obscured by the inherent variability in surgical data. According to the principles of deliberate practice, skill acquisition progresses at a diminishing rate as expertise increases, eventually reaching a plateau of mastery [25].

Several determinants influence the trajectory of a surgeon's learning curve. External factors include technological advancements and evolving clinical guidelines, while intrateam consistency and experience further modulate outcomes. Surgeon-specific attributes—such as innate aptitude, motivation, prior training, and adaptability to new techniques—also significantly impact the rate of skill acquisition and procedural competence [31].

To integrate surgical experience and proficiency into models of difficulty and perioperative risk assessment, the learning curve can be stratified into discrete stages based on case volumes (eg, 0–20 cases, 21–50 cases, etc.). Such stratification incorporates surgeon expertise into risk estimation models, enabling an evaluation of relative reductions in procedural complexity and perioperative risk over time. Additionally, this framework facilitates the identification of procedures suitable for educational and training purposes.

#### 5.1.1 Strengths and implications of the present study

Our study has the potential to positively influence perioperative management in several ways. Firstly, an accurate surgical difficulty and risk prediction system allows for the identification of high-risk patients and better decision-making, improving surgical planning and stratification of individual procedures. This approach enhances awareness of difficult perioperative conditions (both organ specific and non–organ specific) for surgeons, anaesthesiologists, and junior doctors, ultimately improving overall unit preparedness and patient care quality.

Secondly, targeted organ-specific factors—considering the type of surgical approach (open, laparoscopic, or robotic)—could form the foundation of a comprehensive checklist. This could serve as a decision-making tool for surgeons, further assisting in surgical planning and identifying procedures that are suitable for teaching purposes.

#### 5.1.2 Impact on clinical practice

The introduction of the CAMUS IPRADES could reinvigorate risk assessment in urological surgery and potentially extend to other surgical specialities. By enhancing the understanding of surgical difficulty and perioperative risks, these indices may optimise the allocation of critical resources.

The insights from this study could provide greater transparency for patient counselling regarding potential surgical morbidity, follow-up care, and communication with families, improving the accuracy and quality of patient consent. Furthermore, fostering competition between surgeons and hospitals through intersurgeon and interhospital comparisons could serve as an opportunity to elevate surgical quality, improve unit efficiency, and ultimately enhance patient care.

Additionally, the creation of an online calculator for daily clinical use would streamline the adoption and routine implementation of the indices. For the tool to reach its full potential, it must be easily accessible, provide timely results, and undergo validation in a multicentric setting.

#### 5.1.3 Impact on research

If implemented effectively, the indices could underpin a wide range of clinical research initiatives, including prospective clinical trials where patients are randomised to receive precautionary measures based on their risk scores. Academic reporting of these trials would allow for comparisons of outcomes between surgeons and hospitals, contributing to improvements in the overall quality of care. With regard to this multilevel study, external validation in a prospective multicentric validation cohort will be performed with a multicentric dataset from high-volume urological centres and are actively exploring collaborations within the CAMUS network and beyond to support this next phase.

#### 5.1.4 Limitations

Despite its strengths, the CAMUS classification and its development face several limitations. Notably, the comprehensive data collection required for calculating the indices in daily practice is time consuming, which may hinder widespread adherence. As with any expert consensus methodology, there is an inherent risk of a selection bias and response variability. Additionally, the lack of randomisation in the Delphi survey questionnaire poses a potential limitation. However, to mitigate these concerns, we have taken several measures to enhance the rigour of our Delphi process. Firstly, we will ensure that the panel comprises a diverse group of high-volume urological surgeons from multiple institutions and geographical regions, thus improving representativeness. Secondly, expert selection will be based on predefined objective criteria, such as surgical experience, academic contributions, and case volume, to reduce the selection bias. Thirdly, response variability will be addressed by using multiple Delphi rounds, allowing for iterative feedback and convergence towards consensus. While we acknowledge that randomisation is not a standard feature of the Delphi methodology, these steps will help minimise biases and improve the reliability of the expert-derived parameters. Furthermore, we consider sensitivity analyses where applicable.

Nevertheless, to the best of our knowledge, this study will be the first to incorporate a broad range of easily accessible pre- and intraoperative parameters into a risk and difficulty stratification tool [32].

For a detailed overview of the strengths and limitations of the study, refer to Table 6.

**Table 6:** Strengths, implications on clinical practice and research, and limitations of the CAMUS IPRADES indices

#	
	*Strengths*
1	Accurate risk prediction allowing for the identification of high-risk patients and improvement of decision-making/indication
2	Surgeon awareness of difficult perioperative conditions (ie, non–organ-specific factors and targeted organ-specific factors) leading to improved surgical planning and stratification of individual surgeries for their appropriateness to be performed as teaching cases
3	Allocation of critical resources depending on risk stratification
4	Awareness of the expected intra- and postoperative courses by surgeons, anaesthesiologists, and junior doctors in the respective fields, ultimately improving doctor and unit preparedness and patient quality of care
5	Intersurgeon and interhospital comparisons stimulating a competition between surgeons to better their surgical outcomes and ultimately improve patient quality of care
6	Potential for the CAMUS IPRADES indices to be introduced in the form of an online calculator, which should be easily accessible and should allow for computation of the scores in a timely matter, and may be used routinely in clinical practice
7	Potential for the targeted organ-specific factors (also dependent on the surgical approach, ie, open vs laparoscopic vs robotic) to become part of a comprehensive dictionary with a checklist for surgeons for decision-making/indication and surgical planning, and again, to stratify for the appropriateness of the individual operations to be performed as teaching cases
	*Implications*
1	Stimulation of a competition between urologists and centres internationally
2	Improvement of surgical quality and unit efficiency
3	Providing a better understanding of surgical difficulty, and peri- and postoperative risk estimation to optimise allocation of critical resources
4	Offering transparency for patient counselling regarding potential surgical morbidity
5	Improvement of accuracy and quality of patient consent
6	Creation of an online calculator for daily practice
7	Creation of an opportunistic window for a wide variety of clinical research, including prospective randomised and nonrandomised trials
8	Academic reporting, allowing for clinical trials and comparison of outcomes between surgeons and between hospitals (with the aim to improve standard quality of care)
	*Limitations*
1	Time consuming procedure of collecting comprehensive data to calculate indices in clinical practice
2	Lack of randomisation in Delphi survey questionnaire

IPRADES = Intra- and Postoperative Risk and Difficulty Estimation Indices.

### 5.2 Conclusion

Emerging evidence suggests that risk assessment plays a crucial role in improving patient outcomes. Preoperative functional evaluation, prediction of intraoperative surgical complexity, and consideration of unforeseen intraoperative events enhance multidisciplinary decision-making, optimise the allocation of critical care resources, improve patient communication, and facilitate outcome comparisons between surgeons and hospitals. Systematic risk documentation is essential, particularly for high-risk patients and those undergoing technically demanding procedures. Once validated and widely accessible, our risk stratification tools could seamlessly be integrated into standard pre- and intraoperative assessments, ultimately enhancing the quality of patient care.

  ***Author contributions:*** Marc A. Furrer had full access to all the data in the study and takes responsibility for the integrity of the data and the accuracy of the data analysis.

  *Study concept and design:* Lawrentschuk, Furrer.

*Acquisition of the data:* Furrer.

*Analysis and interpretation of the data:* Furrer.

*Drafting of the manuscript:* Furrer, Soliman.

*Critical revision of the manuscript of important intellectual content:* All authors.

*Statistical analysis:* Walde.

*Obtaining funding:* Furrer.

*Administrative, technical or material support:* None.

*Supervision:* Furrer.

*Other:* None.

  ***Financial disclosures:*** All authors certify that all conflicts of interest, including specific financial interests and relationships and affiliations relevant to the subject matter or materials discussed in the manuscript (e.g. employment/affiliation, grants or funding, consultancies, honoraria, stock ownership or options, expert testimony, royalties, or patents filed, received, or pending), are the following: None.

  ***Funding/Support and role of the sponsor:*** None.
