# Supplementary material for: Development of the CAMUS Intra- and Postoperative Risk and Difficulty Estimation Indices Risk Prediction Tool for Estimating Peri- and Postoperative Outcomes, Including Surgical Difficulty, in Major Urological Surgery—A Protocol for a Delphi Study
Source: Eur Urol Open Sci. 2025 Apr 28;76:23–37. doi: 10.1016/j.euros.2025.04.002 (PMC12434987; doi:10.1016/j.euros.2025.04.002)
Supplement: Supplementary Data 1 [file mmc1.docx]

**Supplementary Table 3: Targeted organ-specific factors (organ-specific scoring system)**

Table 6 provides examples of targeted organ-specific factors (as part of the organ-specific scoring system) when performing radical cystectomy partial or radical nephrectomy, nephroureterectomy and retroperitoneal lymph node dissection.

**Prostatectomy:**

| ***Expected / known (preoperative)*** | | | | ***Unexpected / unknown (intraoperative)*** | | | |
| --- | --- | --- | --- | --- | --- | --- | --- |
| **Parameter** | **Surgeon** | | **Anaesthetist** | **Parameter** | **Surgeon** | | **Anaesthetist** |
|  | **Surgical difficulty**  **(0=no, 1=yes)** | **Risk of complication**  **(0=no, 1=yes)** | **Risk of complication**  **(0=no, 1=yes)** |  | **Surgical difficulty**  **(0=no, 1=yes)** | **Risk of complication**  **(0=no, 1=yes)** | **Risk of complication**  **(0=no, 1=yes)** |
| Size (cc) |  |  |  | Narrow pelvis |  |  |  |
| Numbers of preoperative biopsies |  |  |  | Intrapelvic fatty tissue |  |  |  |
| Numbers of cores taken |  |  |  | Fibrosed tissue (application of Hem-o-loks / metal clips) |  |  |  |
| Type of biopsy (TRUS vs TP) |  |  |  | Adherent tissue in posterior plane |  |  |  |
| Gleason score |  |  |  | Locally advanced / infiltrative tumour |  |  |  |
| PI-RADS |  |  |  |  |  |  |  |
| T stage |  |  |  |  |  |  |  |
| Localisation / size of tumour |  |  |  |  |  |  |  |
| Time between biopsy and surgery (e.g. 1-3 weeks, 3-6 weeks, >6 weeks) |  |  |  |  |  |  |  |
| Previous radiation (inc. dosage, e.g. LDR/HDR) +/- ADT |  |  |  |  |  |  |  |
| Etc. |  |  |  |  |  |  |  |

**Cystectomy:**

| ***Expected / known (preoperative)*** | | | | ***Unexpected / unknown (intraoperative)*** | | | |
| --- | --- | --- | --- | --- | --- | --- | --- |
| **Parameter** | **Surgeon** | | **Anaesthetist** | **Parameter** | **Surgeon** | | **Anaesthetist** |
|  | **Surgical difficulty**  **(0=no, 1=yes)** | **Risk of complication**  **(0=no, 1=yes)** | **Risk of complication**  **(0=no, 1=yes)** |  | **Surgical difficulty**  **(0=no, 1=yes)** | **Risk of complication**  **(0=no, 1=yes)** | **Risk of complication**  **(0=no, 1=yes)** |
| Previous BCG (number of cycles / MMC, epirubicin?) |  |  |  | Locally advanced / infiltrative tumour |  |  |  |
| Previous neoadjuvant chemotherapy etc |  |  |  | Fibrosed / adherent tissues |  |  |  |
| cT stage bimanual palpation |  |  |  |  |  |  |  |
| cT stage biopsy / imaging |  |  |  |  |  |  |  |
| Etc. |  |  |  |  |  |  |  |

**Nephrectomy / nephroureterectomy:**

| ***Expected / known (preoperative)*** | | | | ***Unexpected / unknown (intraoperative)*** | | | |
| --- | --- | --- | --- | --- | --- | --- | --- |
| **Parameter** | **Surgeon** | | **Anaesthetist** | **Parameter** | **Surgeon** | | **Anaesthetist** |
|  | **Surgical difficulty**  **(0=no, 1=yes)** | **Risk of complication**  **(0=no, 1=yes)** | **Risk of complication (0=no, 1=yes)** |  | **Surgical difficulty**  **(0=no, 1=yes)** | **Risk of complication**  **(0=no, 1=yes)** | **Risk of complication (0=no, 1=yes)** |
| Radius of the tumour |  |  |  | Locally advanced / infiltrative tumour |  |  |  |
| Exo- vs endo- phytic mass |  |  |  | Fibrosed / adherent tissue |  |  |  |
| Location (anterior vs posterior; and relative to polar lines) |  |  |  |  |  |  |  |
| Hilar tumour |  |  |  |  |  |  |  |
| Etc. |  |  |  |  |  |  |  |

**Retroperitoneal lymph node dissection (RPLND):**

| ***Expected / known (preoperative)*** | | | | ***Unexpected / unknown (intraoperative)*** | | | |
| --- | --- | --- | --- | --- | --- | --- | --- |
| **Parameter** | **Surgeon** | | **Anaesthetist** | **Parameter** | **Surgeon** | | **Anaesthetist** |
|  | **Surgical difficulty**  **(0=no, 1=yes)** | **Risk of complication**  **(0=no, 1=yes)** | **Risk of complication (0=no, 1=yes)** |  | **Surgical difficulty**  **(0=no, 1=yes)** | **Risk of complication**  **(0=no, 1=yes)** | **Risk of complication (0=no, 1=yes)** |
| Recurrent or primary tumour |  |  |  | Quality of tissue planes (e.g., fibrosed / adherent tissue) |  |  |  |
| Template (uni- vs bilateral, and left- vs right-sided) |  |  |  | Unexpected encasement of great vessels |  |  |  |
| Indication (for testicular vs upper urinary tract cancer) |  |  |  |  |  |  |  |
| Size of retroperitoneal mass or masses |  |  |  |  |  |  |  |
| Extent and distribution of retroperitoneal disease |  |  |  |  |  |  |  |
| Relationship of retroperitoneal disease to great vessels |  |  |  |  |  |  |  |
| Preoperative chemotherapy |  |  |  |  |  |  |  |
| Number of lines of chemotherapy preceding surgery |  |  |  |  |  |  |  |
| Degree of size decrease after chemotherapy |  |  |  |  |  |  |  |
| Involvement of multiple viscera |  |  |  |  |  |  |  |
