# Supplementary material for: Development of the CAMUS Intra- and Postoperative Risk and Difficulty Estimation Indices Risk Prediction Tool for Estimating Peri- and Postoperative Outcomes, Including Surgical Difficulty, in Major Urological Surgery—A Protocol for a Delphi Study
Source: Eur Urol Open Sci. 2025 Apr 28;76:23–37. doi: 10.1016/j.euros.2025.04.002 (PMC12434987; doi:10.1016/j.euros.2025.04.002)
Supplement: Supplementary Data 2 [file mmc2.docx]

| Assessment of perioperative risk factors during robot-assisted radical prostatectomy | | | | | | | | | | | | | | |
| --- | --- | --- | --- | --- | --- | --- | --- | --- | --- | --- | --- | --- | --- | --- |
| **Supplementary Table 1a: Delphi round 1 – Identification and description of parameters which impact on perioperative risk**  ***‘Which parameters determine perioperative risk and subsequent risk of morbidity [yes vs no], and if appropriate to what degree (easiest – most difficult; do they impact on peri- and postoperative morbidity and mortality?)’***  ***‘Please find parameters which impact perioperative risk and describe these according to their grade of severity (Grade 1-5,*** *e.g. BMI: 18-22 Grade 1, 23-25 Grade 2, 25-30 Grade 3, 30-40 Grade 4, > 40 Grade 5)*  *Note: Several descriptive circumstances per field is possible* | | | | | | | | | | | | | | |
| **Parameters** | **Grade of severity** | | | | | | | | | | | | | |
| *** | **Impact on severity/risk - Yes (1) vs No (0)** | **Grade 1** | | | **Grade 2** | | | **Grade 3** | | | **Grade 4** | | | **Grade 5** |
| ***Expected / known (preoperative) conditions*** | | | | | | | | | | | | | | |
| BMI (kg/m2) | 1 | 18-22 | | 23-25 | | | 25-30 | | | 30-40 | | | >40 | |
| Diabetes mellitus | 1 | Well controlled, HbA1c <6% | | Adequately controlled, HbA1c 6-7% | | | Inadequately controlled, HbA1c 7-8% | | | Poorly controlled HbA1c >9% | | | Very poorly controlled, HbA1c >12 % | |
| ASA class | 1 | ASA 1 | | ASA 2 | | | ASA 3 | | | ASA 4 | | | ASA 5 | |
| Non-age adjusted Charlson Comorbidity Index | 1 | 0-2 | | 3-4 | | | 5-6 | | | 7-8 | | | >8 | |
| Timing of surgery (AM, PM, after hours before midnight, after hours after midnight) | 1 | AM | | PM | | | After hours, before midnight | | | After hours, after midnight | | | After hours > 3 AM | |
| Prior abdominal surgical history + type of surgery (e.g. bowel resection vs cholecystectomy) | 1 | Explorative laparoscopy incl cholecystectomy and appendectomy | | Explorative open surgery | | | Laparoscopic procedures with resection of tissue (e.g., bowel anastomosis) or mesh insertion | | | Multiple open interventions or laparoscopic procedures with mesh insertion | | | Multiple open interventions with mesh insertion | |
| Surgical approach (e.g. open vs lap vs robot) | Etc. |  | |  | | |  | | |  | | |  | |
| Anti-platelet/anti-coagulant agents (e.g. aspirin vs “other than aspirin” |  |  | |  | | |  | | |  | | |  | |
| Elective vs. emergency |  |  | |  | | |  | | |  | | |  | |
| Previous therapy: non-organ-specific (e.g. radiation) |  |  | |  | | |  | | |  | | |  | |
| Recent MACE (e.g. MI, LE <90d) |  |  | |  | | |  | | |  | | |  | |
| Nutrition status (e.g. albumin) |  |  | |  | | |  | | |  | | |  | |
| Age |  |  | |  | | |  | | |  | | |  | |
| Gender |  |  | |  | | |  | | |  | | |  | |
| Steroid use for chronic condition |  |  | |  | | |  | | |  | | |  | |
| Significant smoking status (e.g. 40-50 PYH) |  |  | |  | | |  | | |  | | |  | |
| Etc. |  |  | |  | | |  | | |  | | |  | |
| ***Unexpected / unknown (intraoperative) conditions’*** | | | | | | | | | | | | | | |
| Estimated blood loss *(e.g. > 500mL*) | 1 | <100ml | | 100-500ml | | | 500-1000ml | | | 1000-1500ml | | | >1500 | |
| Duration of operation *(e.g. > 200min)* | 1 | <1h | | 1-3h | | | 3-6h | | | 6-10h | | | >10h | |
| ECG abnormalities *(e.g. ST elevation)* | Etc. |  | |  | | |  | | |  | | |  | |
| Ventilation / airway difficulties (e.g. high pressures) |  |  | |  | | |  | | |  | | |  | |
| Intraoperative fluids administered *(e.g. > 1.5L*) |  |  | |  | | |  | | |  | | |  | |
| Damage to surrounding organs (e.g., vessel injury, bowel perforation) |  |  | |  | | |  | | |  | | |  | |
| Vasopressors [ug] |  |  | |  | | |  | | |  | | |  | |
| Mean MAP [mmHg] |  |  | |  | | |  | | |  | | |  | |
| Mean heart rate [BPM] |  |  | |  | | |  | | |  | | |  | |
| Etc. |  |  | |  | | |  | | |  | | |  | |
| ***Unexpected / unknown (postoperative) conditions prior to discharge or max within 24h***  ***D*** | | | | | | | | | | | | | | |
| Acute kidney injury *(e.g. GFR decrease by 10 ml/min)* | 1 | | GFR decrease by <25% | | | GFR decrease by 25% | GFR decrease by 50% | | GFR decrease by 75% | | | GFR decrease by >75% | | |
| Significant demand for analgesia *(opioids > 50ug)* | Etc. | |  | | |  |  | |  | | |  | | |
| Haemoglobin decrease preop and POD1 [in g/dl] |  | |  | | |  |  | |  | | |  | | |
| Etc. |  | |  | | |  |  | |  | | |  | | |

| **Supplementary Table 1b: Delphi round 2 – Definition of range of importance/severity of parameters impacting perioperative risk developed in Delphi round 1**  ***‘How important would you rate the following parameters regarding their impact on perioperative risk and subsequent risk of morbidity on a scale 0-10,*** *e.g. Very poorly controlled, HbA1c >12 %: 8; Inadequately controlled, HbA1c 7-8%: 4; Well controlled, HbA1c <6%: 0.*  *Note: point values are [0, 0.5, 1, 1.5,2 etc]* | | | |
| --- | --- | --- | --- |
| **Parameters** | **Complexity**  0  10 | | |
| ***Expected / known (preoperative) conditions*** | | | |
| Diabetes mellitus | | | |
| Well controlled, HbA1c <6% | 0 |  | 10 |
| Adequately controlled, HbA1c 6-7% | 0 |  | 10 |
| Inadequately controlled, HbA1c 7-8% | 0 |  | 10 |
| Poorly controlled, HbA1c >9% | 0 |  | 10 |
| Very poorly controlled, HbA1c >12 % | 0 |  | 10 |
| ***Unexpected / unknown (intraoperative) conditions*** Duration of operation | | | |
| <1h | 0 |  | 10 |
| 1-3h | 0 |  | 10 |
| 3-6h | 0 |  | 10 |
| 6-10h | 0 |  | 10 |
| >10h | 0 |  | 10 |
| ***Unexpected / unknown (postoperative) conditions prior to discharge or max within 24h*** Acute kidney injury | | | |
| GFR decrease by <25% | 0 |  | 10 |
| GFR decrease by 25% | 0 |  | 10 |
| GFR decrease by 50% | 0 |  | 10 |
| GFR decrease by 75% | 0 |  | 10 |
| GFR decrease by >75% | 0 |  | 10 |
