# Supplementary material for: Development of the CAMUS Intra- and Postoperative Risk and Difficulty Estimation Indices Risk Prediction Tool for Estimating Peri- and Postoperative Outcomes, Including Surgical Difficulty, in Major Urological Surgery—A Protocol for a Delphi Study
Source: Eur Urol Open Sci. 2025 Apr 28;76:23–37. doi: 10.1016/j.euros.2025.04.002 (PMC12434987; doi:10.1016/j.euros.2025.04.002)
Supplement: Supplementary Data 3 [file mmc3.docx]

| Assessment of surgical difficulty during robot-assisted radical prostatectomy | | | | | | | | | | |
| --- | --- | --- | --- | --- | --- | --- | --- | --- | --- | --- |
| **Supplementary Table 2a : Delphi Round 1 – Identification and description of parameters which impact on surgical difficulty**  ***‘Which parameters determine surgical difficulty (yes vs no), and if appropriate, to what extent (i.e. how difficult are they? [easiest – most difficult])’***  ***‘Please find parameters which impact surgical difficulty and describe these according to their grade of difficulty (easiest condition – most difficult condition),*** *e.g. bleeding tendency – easiest: totally dry; intermediate easy: minor ooze; standard: moderate ooze; intermediate difficult: significant ooze/bleeding not impacting vision; most difficult: bleeding impacting vision*  *Note: Several descriptive circumstances per field is possible* | | | | | | | | | | |
| **Parameters** | **Grade of difficulty** | | | | | | | | | |
| *** | **Impact on difficulty - Yes (1) vs No (0)** | **Easiest** | | **Moderately easy** | | **Standard** | **Moderately difficult** | | | **Most difficult** |
| ***Expected / known (preoperative) general conditions*** | | | | | | | | | | |
| BMI (kg/m2) | 1 | | 18-22 | | 23-25 | 25-30 | | 30-40 | >40 | |
| Previous surgery *(other than targeted organ, please specify – e.g. open unilateral hernia repair with mesh, or lap LAR etc)* | 1 | | Explorative laparoscopy incl cholecystectomy and appendectomy | | Explorative open surgery | Laparoscopic procedures with resection of tissue (e.g., bowel anastomosis) or mesh insertion | | Multiple open interventions or, laparoscopic procedures with mesh insertion | Multiple open interventions with mesh insertion | |
| Previous non-surgical therapy/interventions: non-organ-specific *(e.g. radiation of operative field)* | 1 | |  | | Antibiotic treatment for prostatitis | Chemo- or immunotherapy | | Multiple prostate biopsies (e.g., during active surveillance) | Radiation therapy in the operative field (dosage according to curative intent) | |
| ASA class | 0 | |  | |  |  | |  |  | |
| Timing of surgery (AM, PM) | 0 | |  | |  |  | |  |  | |
| Etc. |  | |  | |  |  | |  |  | |
| ***Expected / known (preoperative) targeted organ-specific factors*** | | | | | | | | | | |
| Prostate size on MRI (cc) | 1 | | 15-30 | | 31-50 | 51-100 | | 100-200 | >200 or <15 | |
| Presence of middle lobe on MRI | 1 | | - | | Small middle lobe | Intermediate middle lobe | | Large middle lobe | Large middle lobe extending very close to the ureteric orifice | |
| Previous treatment *(e.g. previous radiation (HDR/LDR) +/- ADT)* | 1 | | Antibiotic treatment for prostatitis | | Chemo- or immunotherapy | ADT | | Radiotherapy to the prostate | ADT and radiotherapy to the prostate | |
| Previous surgery *(e.g. Laser-TUR-P or BNI)* | 1 | | Flexible cystoscopy | | Bladder neck incision | Multiple prostate biopsies (e.g., during active surveillance) | | TUR-P or multiple BNIs | Multiple TUR-P | |
| “Operative field fat stranding” on imaging | Etc. | |  | |  |  | |  |  | |
| Size of tumour on MRI *(maximal diameter in cm)* |  | |  | |  |  | |  |  | |
| Associated anatomical difficulties / findings |  | |  | |  |  | |  |  | |
| Size of tumour on MRI *(maximal diameter in cm)* |  | |  | |  |  | |  |  | |
| Numbers of preoperative biopsies |  | |  | |  |  | |  |  | |
| Numbers of biopsy cores taken |  | |  | |  |  | |  |  | |
| Type of biopsy *(TRUS vs TP)* |  | |  | |  |  | |  |  | |
| Gleason score (on biopsy) |  | |  | |  |  | |  |  | |
| Previous TUR-P |  | |  | |  |  | |  |  | |
| cT stage *(DRE / MRI, e.g. organ confined vs locally advanced disease)* |  | |  | |  |  | |  |  | |
| Etc. |  | |  | |  |  | |  |  | |
| ***Unexpected / unknown (intraoperative) conditions***  ***Overall intraoperative conditions*** | | | | | | | | | | |
| Tissue planes (e.g. concrete) | 1 | | Very easily identifiable and dissectible | | Appropriately identifiable and dissectible planes | Partly adherent, partly appropriately identifiable and dissectible planes | | Adherent planes throughout | Significantly adherent planes throughout (‘concrete’) | |
| Bleeding propensity (e.g. ooze) | 1 | | No bleeding | | Minor ooze | Moderate bleeding tendency | | Significant bleeding without clotting and intact vision | Significant bleeding with clotting and visual impairment | |
| Tissue quality *(e.g. fibrosed tissue)* | Etc. | |  | |  |  | |  |  | |
| Vision *(e.g. impaired vision due to extensive fat or significant bleeding)* |  | |  | |  |  | |  |  | |
| Anatomical difficulties / findings (e.g. narrow bony pelvis, accessory vessels) |  | |  | |  |  | |  |  | |
| Tumour characteristics *(e.g. locally advanced, infiltrative tumour)* |  | |  | |  |  | |  |  | |
| Tortuosity of pelvic vessels *(for PLND only)* |  | |  | |  |  | |  |  | |
| Inflammatory fat *(stick/toxic fat in operative field)* |  | |  | |  |  | |  |  | |
| Etc. |  | |  | |  |  | |  |  | |
| ***Surgical steps (Targeted organ-specific factors)*** | | | | | | | | | | |
| **Access** (port placement / adhesiolysis; *e.g. extensive dense adhesions)* | Etc. | |  | |  |  | |  |  | |
| **Bladder takedown** *(e.g. Mesh after hernia repair)* |  | |  | |  |  | |  |  | |
| **Prostate defatting** *(e.g. toxic fat)* |  | |  | |  |  | |  |  | |
| **Endopelvic fascial incision** *(e.g. prominent periprostatic vein with high venous pressures/significant venous bleeding)* |  | |  | |  |  | |  |  | |
| **Anterior bladder dissection** *(e.g. thickened detrusor muscle)* |  | |  | |  |  | |  |  | |
| **Posterior bladder dissection** (e.g. TURP, prostate size > 80cc, *r*ectal adhesions/loss of posterior tissue plane/prior rectal surgery/aberrant rectal anatomy, e.g. J pouch) |  | |  | |  |  | |  |  | |
| **Seminal vesicle/vas dissection** *(e.g. calcified / fibrosed seminal vesicles)* |  | |  | |  |  | |  |  | |
| **Posterior plane dissection/development** *(e.g. extensive compressing pararectal fat)* |  | |  | |  |  | |  |  | |
| **Nerve / neurovascular bundle sparing** *(e.g. adherent to prostate, making dissection difficult)* |  | |  | |  |  | |  |  | |
| **Pedicle identification and division** *(e.g. difficulty for application of Hem-o-loks / metal clips as fibrosed)* |  | |  | |  |  | |  |  | |
| **Deep vascular complex division** *(e.g. broad/large DVC with high* *venous pressure and propensity to bleed)* |  | |  | |  |  | |  |  | |
| **Deep vascular complex suture** *(e.g. high-pressure vein/extensive bleeding)* |  | |  | |  |  | |  |  | |
| **Rocco suture** *(e.g. thin Denonvilliers' fascia)* |  | |  | |  |  | |  |  | |
| **Vesicourethral anastomosis** *(e.g. fragile urethral tissue)* |  | |  | |  |  | |  |  | |
| **Anterior reconstruction** *(e.g. osteophyte/ bone spur (symphysis)* |  | |  | |  |  | |  |  | |

| **Supplementary Table 1b: Delphi round 2 – Definition of range of complexity of parameters developed in Delphi round 1**  ***‘How important would you range the following parameters regarding their impact on surgical difficulty and subsequent risk of morbidity on a scale 0-10,*** *e.g. adherent planes (concrete): 9.5, normal planes: 5, easily identifiable planes: 0*  *Note: point values are [0, 0.5, 1, 1.5,2 etc]*  *Below, examples for expected/known targeted organ-specific factors and unexpected unknown conditions* | | | |
| --- | --- | --- | --- |
| **Parameters** | **Complexity**  0  10 | | |
| ***Expected / known (preoperative) Targeted organ-specific factors***  **Prostate size on MRI** | | | |
| 20-30 cc | 0 |  | 10 |
| 31-50 cc | 0 |  | 10 |
| 51-100 cc | 0 |  | 10 |
| [101-200] cc | 0 |  | 10 |
| >200 cc | 0 |  | 10 |
|  | | | |
| Previous surgery | | | |
| Flexible cystoscopy | 0 |  | 10 |
| Bladder neck incision | 0 |  | 10 |
| Multiple prostate biopsies (e.g., during active surveillance) | 0 |  | 10 |
| TUR-P or multiple BNIs | 0 |  | 10 |
| Multiple TUR-P | 0 |  | 10 |
| ***Unexpected / unknown (intraoperative) conditions*** Tissue planes | | | |
| Very easily identifiable and dissectible planes | 0 |  | 10 |
| Appropriately identifiable and dissectible planes | 0 |  | 10 |
| Partly adherent, partly appropriately identifiable and dissectible planes | 0 |  | 10 |
| Adherent planes throughout | 0 |  | 10 |
| Significantly adherent planes throughout (‘concrete’) | 0 |  | 10 |
